# Supplementary material for: Solar UV-B/A radiation is highly effective in inactivating SARS-CoV-2
Source: Sci Rep. 2021 Jul 20;11:14805. doi: 10.1038/s41598-021-94417-9 (PMC8292397; doi:10.1038/s41598-021-94417-9)
Supplement: Supplementary file 1 — Supplementary Figures. [file 41598_2021_94417_MOESM1_ESM.docx]

**Supplementary Information**

**Solar UV–B/A Radiation is Highly Effective in Inactivating SARS-CoV-2**

**Authors:**

Fabrizio Nicastro^1*^, Giorgia Sironi^2^, Elio Antonello^2^, Andrea Bianco^2^, Mara Biasin^3^, John R. Brucato^4^, Ilaria Ermolli^1^, Giovanni Pareschi^2^, Marta Salvati^5^, Paolo Tozzi^4^, Daria Trabattoni^3^, Mario Clerici^6^

**Affiliations:**

1 Italian National Institute for Astrophysics (INAF) – Rome Astronomical Observatory, Rome, Italy

2 Italian National Institute for Astrophysics (INAF) – Brera Astronomical Observatory, Milano/Merate, Italy

3 Department of Biomedical and Clinical Sciences L. Sacco, University of Milano, Milano, Italy

4 Italian National Institute for Astrophysics (INAF) – Arcetri Astrophysical Observatory, Firenze, Italy

5 Regional Agency for Environmental Protection of Lombardia (ARPA Lombardia), Milano, Italy

6 Department of Pathophysiology and Transplantation, University of Milano and Don C. Gnocchi Foundation, IRCCS, Milano, Italy

* E-mail: [fabrizio.nicastro@inaf.it](mailto:fabrizio.nicastro@inaf.it)

Supplementary Figure S1 Main panel: *day-by-day yearly modulation imprinted by the solar mechanism on the intrinsic reproduction number R_0_=3 of the epidemic (black horizontal dashed line), at the three average latitudes of the countries of our N (green curves), T (orange curves) and S (blue curves) groups. Onsets: infra-day solar-pump modulation of R_o_ at the three latitudes on January 1^st^ (left onset) and July 1^st^ (right onset) 2020, as labeled (see Methods for details).*

Supplementary Figure S2 Simulated mean daily mortality curves as a function of time, from 22 January 2020 through April 2021, *with (solid curves) and without (dashed curves) the effect of the Solar-Pump (see Methods for details)*.
